# Enrichment of Cysteine-Containing Peptide by On-Resin Capturing and Fixed Charge Tag Derivatization for Sensitive ESI-MS Detection

**DOI:** 10.3390/molecules25061372

**Published:** 2020-03-18

**Authors:** Remigiusz Bąchor, Oliwia Gorzeń, Anna Rola, Karolina Mojsa, Karolina Panek-Laszczyńska, Andrzej Konieczny, Krystyna Dąbrowska, Wojciech Witkiewicz, Zbigniew Szewczuk

**Affiliations:** 1 Faculty of Chemistry, University of Wroclaw, 50-383 Wroclaw, Poland; oliwia.gorzen1@gmail.com (O.G.); anna.rola@chem.uni.wroc.pl (A.R.); zbigniew.szewczuk@chem.uni.wroc.pl (Z.S.); 2 Department of Molecular Microbiology, Faculty of Biotechnology, University of Wroclaw, 50-383 Wroclaw, Poland; karolina.mojsa@chem.uni.wroc.pl; 3 1st Department and Clinic of Gynecology and Obstetrics, Wroclaw Medical University, 50-368 Wroclaw, Poland; karolina.panek@gmail.com; 4 Wroclaw Medical University, Department of Nephrology and Transplantation Medicine, 50-556 Wroclaw, Poland; andrzej_konieczny@yahoo.com; 5 Institute of Immunology and Experimental Therapy, Polish Academy of Sciences, 53-114 Wroclaw, Poland; Dabrowska@wssk.wroc.pl; 6 Research and Development Center, Regional Specialized Hospital, 51-124 Wroclaw, Poland; witkiewicz@wssk.wroc.pl

**Keywords:** cysteine, thiopeptides, maleimide, mass spectrometry, fixed charge tags, podocin

## Abstract

High complexity of cell and tissue proteomes limits the investigation of proteomic biomarkers. Therefore, the methods of enrichment of some chemical groups of peptides including thiopeptides are important tools that may facilitate the proteomic analysis by reducing sample complexity and increasing proteome coverage. Here, we present a new method of cysteine-containing tryptic peptide enrichment using commercially available TentaGel R RAM resin modified by the linker containing the maleimide group, allowing thiol conjugation. The captured tryptic peptides containing lysine residue were then tagged by 2,4,6-triphenylpyrylium salt to form 2,4,6-triphenylpyridinium derivatives, which increases the ionization efficiency during mass spectrometry analysis. This makes it possible to conduct an ultrasensitive analysis of the trace amount of compounds. The proposed strategy was successfully applied in the enrichment of model tryptic podocin peptide and podocin tryptic digest.

## 1. Introduction

Cysteine, a proteinogenic amino acid containing the thiol group, plays a crucial role in protein structure and functions. It was found that 91% of the known proteins contain at least one cysteine residue and that it is present in more than 24% of predicted tryptic peptides [1]. This makes the cysteine-containing peptides a very attractive target for chemoselective fractionation, which may facilitate proteome analysis using a subset of tryptic peptides. The reactivity of cysteine thiol and thiolate anions including high nucleophilicity and redox chemistry makes this amino acid residue a special easily modified target for electrophilic reagents and thiol-disulfide reagents. 

Several strategies for cysteine-containing peptide capturing have been developed. One of the potential approaches is based on the thiol-disulfide chemistry using thiopropyl sepharose [2]. Briefly, this method assumes the capture of reduced cysteine-containing protein digest with non-alkylated thiols using thiopropyl resin, followed by their release with the reducing agent. Another popular strategy is based on the application of a tag that selectively alkylates reduced cysteine thiols and enhances chromatographic selection [3]. Usually, this can be achieved using an alkylating biotin reagent, followed by the affinity chromatography using the avidin-modified stationary phase. Additionally, Ren et al. proposed a new strategy specifically targeting cysteine-containing peptides based on the derivatization of cysteine residues before enzymatic digestion using a quaternary amine tag and the final enrichment of cysteine-containing peptides by strong cation exchange chromatography [4].

Maleimide derivatives are commonly used alkylating reagents for thiol groups due to the high specificity and reactivity, lack of byproducts, and the stability of the formed thioether product [5]. It was shown that the thiol−maleimide conjugation is used for biomolecule derivatization or to conjugate different compounds [6]. Additionally, the potential application of maleimide groups for biomolecular immobilization using monolayers on various metallic, glass surfaces, and polymeric materials was presented [7,8]. Although the maleimide group has been extensively exploited in biomolecular immobilization of thiol-containing peptides and proteins, the potential applicability of commercially available resins, designed for use in peptide synthesis, and modified with maleimide groups has not been reported yet.

Recently, mass spectrometry has become a method of choice in the rapid analysis of molecules. However, due to the low ionization efficiency of some compounds during the electrospray ionization-mass spectrometry (ESI-MS) experiment, the reliable identification of the trace amount of the substance is often limited. This problem is also related to the analysis of cysteine-containing peptides after their immobilization on modified solid support. Previously, we developed an efficient method of peptide conjugate synthesis containing various *N,N,N*-trialkylglycine moieties that act as ionization enhancers for the analysis of peptides at the attomole (10^−18^ M) level [9]. Although the procedure is useful in combinatorial chemistry [10], its application in peptide sequencing is limited due to Hofmann elimination during tandem mass spectrometry (MS/MS) experiments [11]. To overcome the possibility of this unwanted fragmentation, bicyclic [12] and spiro [13] scaffolds were developed as stable ionization tags, where all bonds susceptible to cleavage are protected in the form of 5- or 6-member ring heterocycles. Recently, we developed new very promising ionization markers based on 2,4,6-triphenylpyridinium and 2,4,6-trimethylpyridinium salts [14,15]. The fixed positive charge of the pyridinium group enhances the ionization efficiency and allows for efficient analysis of the trace amount of peptide. The application of inexpensive and commercially available pyrylium salt as a derivatization reagent allows for selective in-solution derivatization of complex mixtures of peptides, containing lysine residue with a reactive ε-amino group. The 2,4,6-triphenylpyridinium-modified peptides generate an abundant protonated 2,4,6-triphenylpyridinium ion in MS/MS experiments. This fragment was found to be a promising reporter ion for the multiple reactions monitoring (MRM) analysis [16].

In this article, we present the novel strategy of cysteine-containing tryptic peptide enrichment using maleimide-containing TentaGel R RAM resin and the derivatization of lysine residues captured peptides using 2,4,6-triphenylpyrylium salts for sensitive ESI-MS analysis.

## 2. Results and Discussion

**Figure 1:**
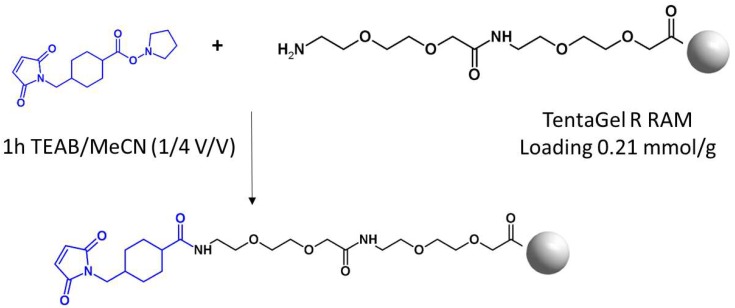
Schematic presentation of the preparation of modified TentaGel R RAM resin for selective capture of cysteine-containing peptides.

The aim of this work was to develop a new method of the enrichment of cysteine-containing tryptic peptide on solid support using commercially available TentaGel R RAM resin modified by the linker containing maleimide moiety, followed by the fixed charge tag derivatization. Such modification increases the ionization efficiency of peptides, making possible their ultrasensitive analysis by ESI-MS [14]. The commercially available TentaGel R RAM resin with the Ring linker was used due to its hybrid and porous structure, allowing swelling in water, penetration by higher mass molecules like enzymes, low capacity, and facile cleavage achieved by 95% trifluoroacetic acid (TFA). The resin was modified by Peg in the form of 9-aza-3,6,12,15-tetraoxa-10-on-heptadecanoic acid (ATHA), which serves as a spacer, providing the distance between the solid support and the reactive group (Figure 1). The amino group of ATHA was modified with *trans*-*N*-succinimidyl 4-(maleimidomethyl)cyclohexane-1-carboxylate (Mal-AMCHC-OSu) in a mixture composed of triethylammonium bicarbonate (TEAB) buffer/acetonitrile (1/4 v/v) for one hour at room temperature.

**Figure 2:**
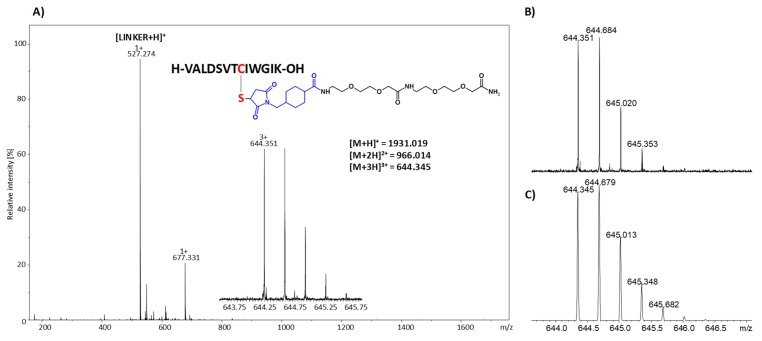
(**A**) Mass spectrum of the obtained product of cysteine-containing podocin tryptic peptide captured using the designed TentaGel resin. The *m/z* values for different ion forms of the expected product were present; (**B**) observed and (**C**) theoretical isotopic pattern of the signal corresponding to the [M + 3H]^3+^ ion of the final product.

The prepared functionalized resin containing the maleimide reactive group was then incubated with model peptides with the H-VALDSVTCIWGIK-OH sequence, which is the tryptic fragment of podocin, the protein that may serve as a preeclampsia biomarker [17]. The reaction was performed in 0.1 M triethylammonium bicarbonate (TEAB) buffer within 1, 3, and 24 h at room temperature. In the first experiment, 0.1 mg of the model peptide was incubated with 20 mg of the functionalized resin and the product of the reaction was cleaved by 95% TFA, lyophilized, and analyzed by mass spectrometry. Peptide concentration used in the experiment was higher than that of the thiopeptide present in the biological samples, however, the main goal of this experiment was to confirm the reaction between the peptide and designed linker located on the resin. The obtained ESI-MS spectrum is presented in Figure 2.

**Figure 3:**
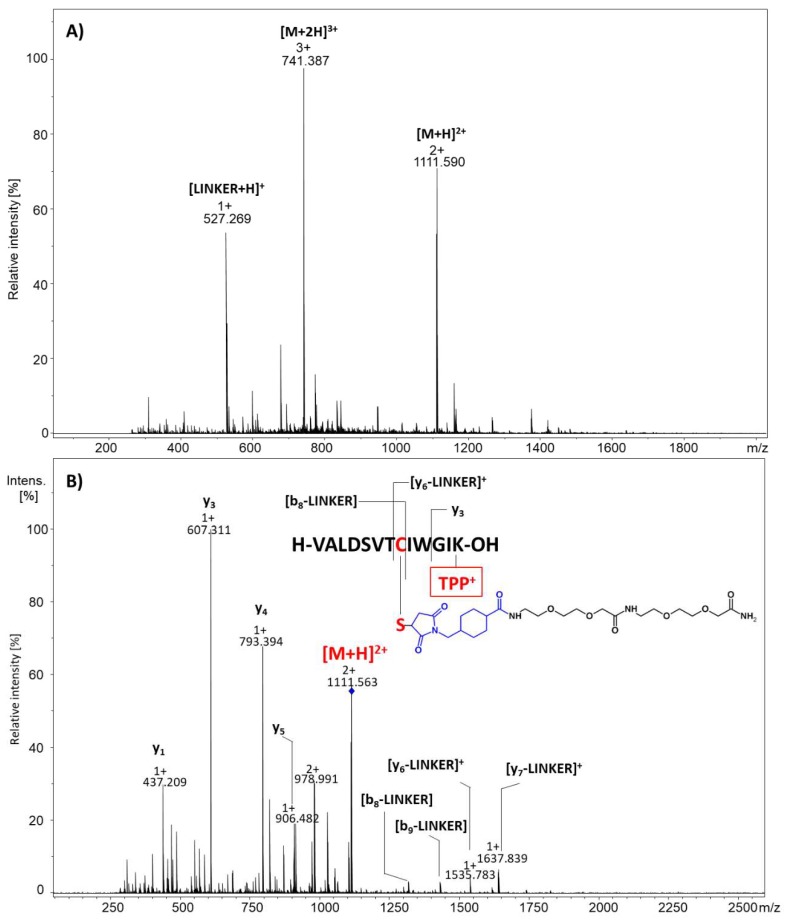
(**A**) ESI-MS spectrum of the obtained peptide conjugate modified by the quaternary ammonium group in the form of the 2,4,6-triphenylpyridinium salt (TPP). (**B**) ESI-MS/MS spectrum of the obtained peptide conjugate modified by TPP. Parent ion *m/z* 1111.074, collision energy 40 eV.

In the obtained ESI-MS spectrum, the most intensive signal corresponded to the protonated ion of the designed linker, composed of the 4-(maleimidomethyl)cyclohexane-1-carboxylate group and ATHA spacer ([LINKER + H]^+^). The low intensive signal corresponding to the [M + 3H]^3+^ ion of the expected product of thiopeptide capture was observed. The isotopic pattern of the signal corresponding to the [M + 3H]^3+^ product of the reaction was compared with the theoretical form confirming their identity (Figure 2B,C). However, due to the low intensity of the signal, it was impossible to confirm the chemical structure of the product using tandem mass spectrometry. The high intensity of the signal corresponding to the linker may result from the applied molar peptide/linker ratio, which was 1/60. Therefore, to increase the ionization efficiency, the captured peptide located on the resin was modified by the quaternary ammonium tag in the form of 2,4,6-triphenylpyrylium salt. The product was then analyzed by ESI-MS and the obtained spectrum is presented in Figure 3A. Highly intensive signals at *m/z* 1111.074 and 741.042 corresponded to the [M + H]^2+^ and [M + 2H]^3+^ ions of the model peptide conjugated with the linker and derivatized with the quaternary ammonium group. The intensity of signals representing the final products was now more than 100 times higher than before derivatization. Additionally, the signal of the unreacted linker was observed (*m/z* 527.259 [LINKER + H]^+^). The signal corresponding to the unmodified peptide with a linker at *m/z* 644.351 was not observed, which clearly indicates the high efficiency of the applied derivatization reaction. To confirm the chemical structure of the obtained peptide conjugate, the tandem mass spectrometry experiment was performed for the ions at *m/z* 1111.074 and the obtained MS/MS spectrum for the [M + H]^2+^ ion is presented in Figure 3B.

The obtained MS/MS spectrum of the ion corresponding to the peptide conjugated with the maleimide-containing linker and modified by TPP salt (Figure 3B) mostly presented y type ions. However, N-terminal fragment ions were also observed (b_8_-LINKER, b_9_-LINKER). The presence of y type ions is related to the location of the fixed charge tag in the form of the quaternary ammonium group at the C-terminal amino acid residue. The fragmentation occurred and resulted in the y-LINKER (y ion type of peptide conjugate with the linker), b-LINKER (b ion type of peptide conjugate with the linker), and y type ion formation. The obtained MS/MS spectrum clearly confirms the chemical structure of the obtained peptide conjugate.

**Figure 4:**
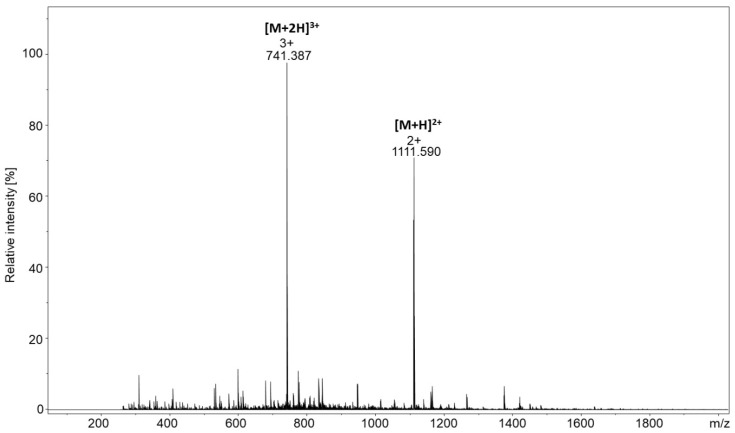
ESI-MS spectrum of the derivatized peptide conjugate obtained from a single resin bead.

To present how sensitive the proposed strategy is, we performed the capturing of the model cysteine-containing peptide with the H-VALDSVTCIWGIK-OH sequence using only one bead of the functionalized TentaGel resin. A total of 0.1 mg of the model peptide solution was incubated for 24 h in 0.1 M TEAB buffer at room temperature and derivatized with the TPP fixed charge tag. The obtained ESI-MS spectrum (Figure 4) shows only the signals corresponding to the captured and derivatized peptide without any signals, indicating the presence of unreacted linker or non-derivatized peptide.

Additionally, to show the selectivity of the proposed method toward the cysteine-containing peptides, the model peptide and its oxidized analogue containing disulfide bridge were incubated with the linker-containing TentaGel resin and derivatized with the fixed charge tag. The obtained results clearly demonstrate that only the cysteine-containing peptide was captured and derivatized, whereas any signals corresponding to other products were not observed (Supplementary Materials, Figure S1).

**Figure 5:**
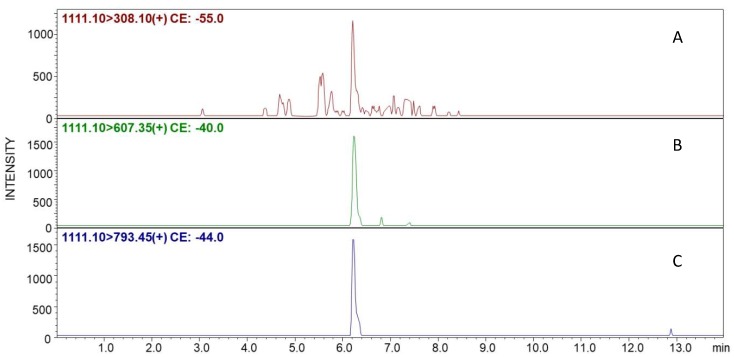
Multiple reaction monitoring (MRM) chromatograms obtained after analysis of podocyte tryptic digest for the following transitions: (**A**) 1111.10→308.10 *m/z* ([TPP + H]^+^), (**B**) 1111.10→607.35 *m/z* (y_3_), and (**C**) 1111.10→793.45 *m/z* (y_4_), corresponding to the fragment ions of peptide conjugated with the functionalized linker and modified by 2,4,6-triphenylpyridinium (TPP) fixed charge tag.

We also tested the applicability of the developed method in a more complex system consisting of the podocyte cell culture obtained as described by Vogelmann and co-workers [18]. Podocytes are highly differentiated cells located in the outer space of the glomerular basement membrane. Their presence in urine refers to podocyturia [19]. The presence of podocytes as well as podocyte-specific proteins (such as nephrin, synaptopodin, and podocin) in the urine of pregnant women suggests an appearance of preeclampsia at an early stage [20]. Podocin showed up as the most robust podocyte marker compared with nephrin and synaptopodin with 100% sensitivity in women with preeclampsia. Therefore, podocin is considered as one of the most podocyte-specific biomarkers of preeclampsia [17]. The aim of this part of our work was to identify the cysteine-containing tryptic fragment of human podocin with the ^247^H-VALDSVTCIWGIK-OH^259^ sequence. The prepared modified resin containing the designed linker with maleimide moiety was incubated with the podocyte tryptic digest followed by TPP. The obtained product was cleaved from the resin in the presence of TFA. The LC-MS analysis was performed using multiple reaction monitoring (MRM) mode. The results are presented in Figure 5. For this purpose, the following MRM transitions were used: 1111.10→308.10 *m/z* ([TPP + H]^+^), 1111.10→607.35 *m/z* (y_3_), and 1111.10→793.45 *m/z* (y_4_). The collision energies for all of the presented transitions were obtained after the automatic MRM method optimization.

The obtained MRM chromatograms present the signals at the retention time of 6.1 min, corresponding to the transitions characteristic for selected parent ions of the investigated peptide conjugate modified by the TPP ionization tag. The performed experiment clearly confirms that the designed maleimide-containing TentaGel R RAM resin captures the thiopeptidem even from the cellular tryptic digest mixture. The observed retention time correlates with the retention time of the model peptide captured by the proposed linker and modified by the ionization tag.

It may be speculated that longer incubation times (24 h) may result in the formation of oxidative modifications of the model peptide, thus reducing the amount of the reactive form of peptide able to react with the linker attached to the resin.

## 3. Materials and Methods

### 3.1. Reagents

All solvents and reagents were used as supplied. Fmoc amino acid derivatives and Fmoc-Lys(Boc)-Wang resin (0.32 mmol/g) were purchased from Novabiochem (Merck, Darmstadt, Germany). *N*-[(Dimethylamino)-1*H*-1,2,3-triazolo-[4,5-*b*]pyridin-1-ylmethylene]-*N*-methylmethanaminium hexafluorophosphate *N*-oxide (HATU), trifluoroacetic acid (TFA), 17-(9-Fluorenylmethyloxycarbonyl-amino)-9-aza-3,6,12,15-tetraoxa-10-on-heptadecanoic acid, and *trans*-N-Succinimidyl 4-(maleimidomethyl)cyclohexane-1-carboxylate (Mal-AMCHC-OSu) were obtained from IrisBiotech (Darmstadt, Germany). Solvents for peptide synthesis (*N,N*-dimethylformamide (DMF), dichloromethane (DCM), (*N*-ethyldiisopropylamine (DIEA)), tetraethylammonium bicarbonate (TEAB), 2,4,6-triphenylpyrylium tetrafluoroborate, 1,4-dithiothreitol (DTT), phenol, thioanisole, and trypsin (TPCK-from bovine pancreas) were obtained from Sigma Aldrich (Saint Louis, MO, USA). Triisopropylsilane (TIS) was from Fluka (Bucharest, Romania). Amicon^®^ Ultra Centrifugal Filters were purchased from Merck (Darmstadt, Germany).

### 3.2. Peptide Synthesis

The synthesis of the model peptide on the Fmoc-Lys(Boc)-Wang resin (loading 0.58 mmol/g) was performed manually in polypropylene syringe reactors (Intavis AG, Tübingen, Germany) equipped with polyethylene filters, according to a standard Fmoc (9-fluorenylmethoxycarbonyl) solid phase synthesis procedure [21]. At the end of the synthesis, the peptidyl–resin was washed with DMF (7 × 1 min), DMF/DCM (1:1; v:v, 1 min), DCM (3 × 1 min), and dried in vacuo. Synthesized peptide was cleaved from the resin and deprotected using modified reagent K (TFA/water/phenol/thioanisole/TIS 82.5/5/5/5/2.5 v/v/v/v/v) at room temperature for 2 h, the resin was rinsed with TFA, and the products were precipitated with cold diethyl ether (Et_2_O). The chemical structure of the synthesized compound was determined by ESI-MS/MS analysis.

### 3.3. Preparation of Modified TentaGel MB RAM Resin

The synthesis of the capturing linker was performed manually in polypropylene syringe reactors (Intavis AG) equipped with polyethylene filters on the TentaGel MB RAM resin (loading 0.28 mmol/g). The spacer in the form of 17-(9-Fluorenylmethyloxycarbonyl-amino)-9-aza-3,6,12,15-tetraoxa-10-on-heptadecanoic acid was attached to the resin, according to the standard Fmoc solid phase synthesis procedure [21]. Then, the spacer-containing resin was dried. The dried resin was then swallowed in water (30 min) and washed with TEAB buffer (3 × 1 min). The Mal-AMCHC-OSu (5 eq) was dissolved in the mixture composed of TEAB/acetonitrile (1/4 v/v) and added to the syringe. The mixture was incubated at room temperature for 1 h, washed with acetonitrile (3 × 1 min), and the ninhydrin test was performed to check the efficiency of the acylation of the amino groups. Such resin was directly used for peptide capturing.

### 3.4. Peptide Capturing by the Modified TentaGel Resin

The obtained TentaGel resin containing the spacer and peptide capturing group was incubated with the peptide dissolved in the mixture composed of TEAB/acetonitrile (1/4, v/v) at room temperature for 24 h. Then, the solution was removed and the resin was washed with the TEAB/acetonitrile (1/4, v/v) mixture (3 × 1 min), H_2_O (3 × 1 min), H_2_O/MeOH (1 × 1 min), MeOH (3 × 1 min), and dried in vacuo. The product of the reaction was cleaved by incubation of the modified resin with the mixture of TFA/H_2_O/TIS (95/0.25/0.25 v/v/v) for 2 h at room temperature, evaporated under nitrogen stream, lyophilized, and analyzed by mass spectrometry.

### 3.5. Derivatization by 2,4,6-Triphenylpyrylium Tetrafluoroborate

The dry modified TentaGel resin with the captured peptide was incubated in DMF (30 min). Then, the mixture containing 2,4,6-triphenylpyrylium tetrafluoroborate (5 eq) and TEA (5 eq) was added and incubated for 3 h at 60 °C. Then, the resin was washed with DMF (7 × 1 min), DMF/DCM (1 × 1 min, v/v), DCM (3 ×1 min), DCM/MeOH (1 × 1 min, v/v), MeOH (3 × 1 min), and dried in vacuo. The product was cleaved from the resin by incubation of the modified resin with the mixture of TFA/H_2_O/TIS (95/0.25/0.25 v/v/v) for 2 h at room temperature, lyophilized, and analyzed by mass spectrometry.

### 3.6. Preparation of the Podocyte Sample

For the experiment, 30,000 podocytes were used. The sample was suspended in 1 mL of 0.1 M TEAB buffer containing 0.1% of RapiGest and sonicated for 30 min. The resulting mixture was transferred into an Amicon^®^Ultra centrifugal filter and centrifuged (4000 rpm for 20 min, 30 °C, Sigma Aldrich, Saint Louis, MO, USA). Then, the sample was washed twice using 0.1 M TEAB and centrifuged in the same conditions to remove the low-molecular compounds. In the next step, the sample was incubated with DTT (100 mL, 0.2 M) over 40 min in room temperature followed by washing (3 × 1 mL of 0.1 M TEAB) in an Amicon^®^Ultra centrifugal filter. Similarly, the mixture was washed three times with 0.1 M TEAB in order to remove the reagents. The obtained supernatant was placed into an Eppendorf tube. Then, 50 μg of trypsin was added in 200 μL of 0.1 M TEAB and the sample was incubated at 37 °C overnight. After digestion, 20 μL of formic acid was added and the sample was lyophilized.

### 3.7. Purification

The synthesized peptide was purified using the analytical HPLC Thermo Separation system (Thermo Fisher Scientific, Waltham, MA, USA ) with UV detection (210 nm) with a YMC-Pack RP C18 column (4.6 × 250 mm, 5 μm), with a gradient elution of 0–40% *B* in *A* (*A* = 0.1% TFA in water; *B* = 0.1% TFA in acetonitrile/H_2_O, 4/1, v/v) over 30 min (flow rate 1 mL/min). The main fraction, corresponding to the peptide, was collected and lyophilized.

### 3.8. Mass Spectrometry

All ESI-MS experiments were performed on a micrOTOF-Q mass spectrometer (Bruker Daltonics, Bremen, Germany) equipped with a standard ESI source. The instruments were operated in the positive-ion mode and calibrated with the Tunemix™ mixture (Agilent Technologies, Palo Alto, CA, USA). The mass accuracy was better than 5 ppm. Analyte solutions (70 μL) were introduced at a flow rate of 3 μL/min. The instrument parameters were as follows: for micrOTOF-Q MS: scan range: 50–3000 *m/z*; drying gas: nitrogen; flow rate: 4.0 L/min, temperature: 200 °C; potential between the spray needle and the orifice: 4.2 kV.

### 3.9. Collision-Induced Dissociation

All MS/MS experiments were performed manually in data independent mode. The singly and doubly protonated precursor ions were selected on the quadrupole and subsequently fragmented in the hexapole collision cell. For one MS/MS experiment, only one precursor ion was isolated and fragmented. Argon was used as a collision gas. The obtained fragments were registered as an MS/MS (tandem mass spectrometry) spectrum. The collision energy (10–30 V) was optimized for the best fragmentation. For MS spectra analysis, Bruker Compass DataAnalysis 4.0 software was used.

### 3.10. Liquid Chromatography-Mass Spectrometry (LC-MS) Analysis in Multiple Reaction Monitoring (MRM) Mode

Liquid Chromatography-Mass Spectrometry analysis in multiple reaction monitoring mode (LC-MS/MRM) experiments were performed on a LCMS-8050 Shimadzu apparatus (Shimadzu Corporation, Kyoto, Japan), with a UHPLC Nexera X2 system, equipped with an Aeris Peptide XB-C18 column (50 mm × 2.1 mm) 3.6 μm bead diameter, equilibrated at 27 °C. The LC system was operated with the mobile phase, consisting of solvent A: 0.1% formic acid in H_2_O and solvent B: 0.1% formic acid in MeCN. The gradient conditions (B%) were from 5 to 80% B within 13 min. The flow rate was 0.3 mL/min and the injection volume was 5 μL. The MRM method was optimized automatically and the following transitions were chosen: 1111.10→308.10 *m/z* ([TPP + H]^+^), 1111.10→607.35 *m/z* (y_3_), and 1111.10→793.45 *m/z* (y_4_). For MRM data analysis, LabSolutions software, version 3.0 was used.

The experimental procedures were conducted in accordance with the ethical standards of the Helsinki Declaration and were approved by the Local Bioethical Commission No. KP/No. 2/year 2015, regarding the project “Searching for new diagnostic methods in pre-eclampsia”.

## 4. Conclusions

In conclusion, we demonstrated a new method of cysteine-containing tryptic peptide enrichment using commercially available TentaGel R RAM resin modified by the linker containing a thiol-reactive maleimide group. The captured peptides containing C-terminal lysine residues were modified at the ε-amino group by the quaternary ammonium group in the form of 2,4,6-triphenylpyrylium salt forming 2,4,6-triphenylpyridinium derivatives. This methodology allowed for the sensitive detection of cysteine-containing podocin tryptic peptide, which could be used as a potential method in the investigation of biomarkers of preeclampsia.

## Supplementary Materials

The following are available online, Figure S1: ESI-MS spectrum obtained after incubation of model cysteine-containing peptide and its oxidized analogue containing disulphide bridge. The captured compound was derivatized by TPP before the cleavage from the resin. Signals at m/z 741.387 and 1111.590 corresponds to the captured and TPP-derivatized VALDSVTCIWGIK peptide ions with 2+ and 3+ charge. Additional signals corresponding to its oxidized form were not identified.
