# Supplementary material for: Enrichment of Cysteine-Containing Peptide by On-Resin Capturing and Fixed Charge Tag Derivatization for Sensitive ESI-MS Detection
Source: Molecules. 2020 Mar 18;25(6):1372. doi: 10.3390/molecules25061372 (PMC7144375; doi:10.3390/molecules25061372)
Supplement: Supplementary file 1 [file molecules-25-01372-s001.pdf]

Supplementary data

Enrichment of cysteine-containing peptide by on-resin capturing and fixed charge tag derivatization for sensitive ESI-MS detection

Remigiusz Bąchor[1]*, Oliwia Gorzeń[1], Anna Rola[1], Karolina Mojsa[1], Karolina Panek-Laszczyńska[2], Andrzej konieczny[3], Krystyna Dąbrowska[4,5], Wojciech Witkiewicz[5], Zbigniew Szewczuk[1]

[1] Faculty of Chemistry, University of Wroclaw, Wroclaw, Poland
[2] 1st Department and Clinic of Gynaecology and Obstetrics, Wroclaw Medical University, Wroclaw, Poland
[3] Wroclaw Medical University, Department of Nephrology and Trasplantation Medicine, Wrocław, Poland
[4] Institute of Immunology and Experimental Therapy, Polish Academy of Sciences, Wroclaw, Poland
[5] Research and Development Center, Regional Specialized Hospital, Wroclaw, Poland

Corresponding Author

* Remigiusz Bąchor, Faculty of Chemistry, University of Wrocław, F. Joliot-Curie 14, 50-383 Wrocław, Poland, Fax: +48 71 328 2348, Tel.: +48 71 375 7218, E mail: remigiusz.bachor@chem.uni.wroc.pl

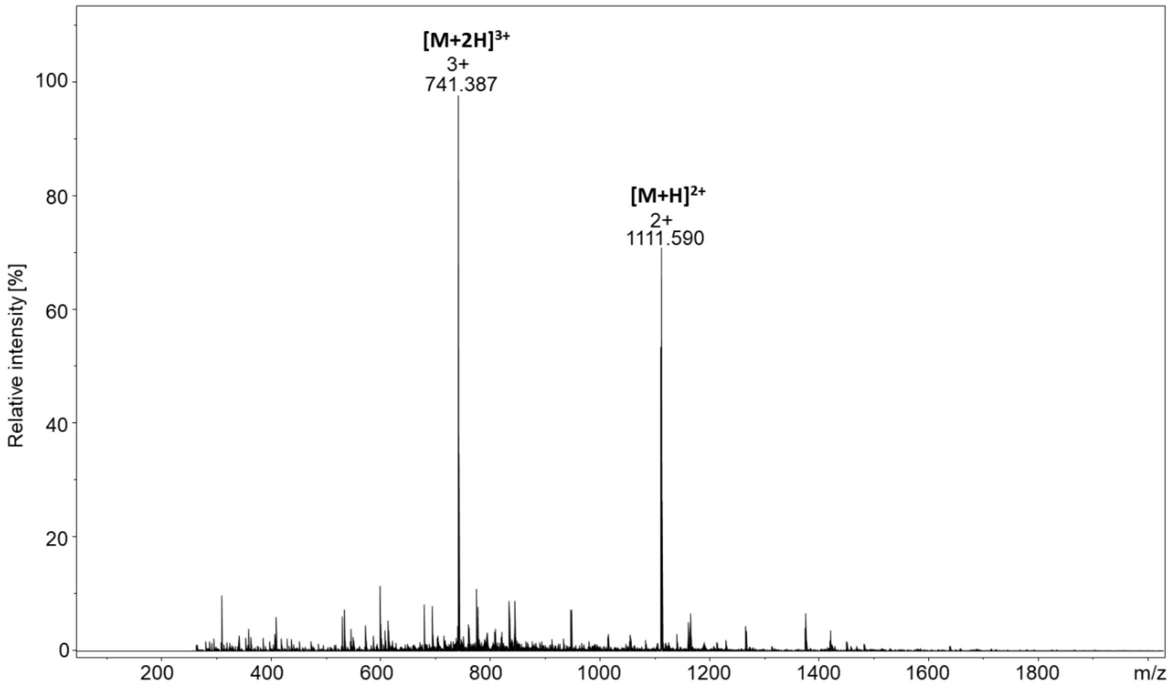

Figure 1S. ESI-MS spectrum obtained after incubation of model cysteine-containing peptide and its oxidized analogue containing disulphide bridge. The captured compound was derivatized by TPP before the cleavage from the resin. Signals at *m/z* 741.387 and 1111.590 corresponds to the captured and TPP-derivatized VALDSVTCIWGIK peptide ions with 2+ and 3+ charge. Additional signals corresponding to its oxidized form were not identified.
